# Relationship between nailfold capillaroscopy findings and the etiology and prognosis of interstitial lung disease

**DOI:** 10.1007/s10067-024-07049-5

**Published:** 2024-07-04

**Authors:** Şerife Şeyda Zengin Acemoğlu, İpek Türk, Pelin Pınar Deniz, Mehmet Ali Aşık, Didem Arslan, İsmail Hanta, İlker Ünal

**Affiliations:** 1 Department of Internal Medicine, Division of Rheumatology, FacultyofMedicine, Cukurova University, Adana, Turkey; 2 Department of Chest Diseases, FacultyofMedicine, Cukurova University, Adana, Turkey; 3 Department of Biostatistic, FacultyofMedicine, Cukurova University, Adana, Turkey

**Keywords:** Connective tissue-associated interstitial lung diseases, Interstitial lung disease, Microvascular damage, Nailfold capillaroscopy

## Abstract

**Objectives:**

Connective tissue-associated interstitial lung diseases (CTD-ILD) are believed to be caused by microvascular damage. The objective of this study was to assess the nailfold capillaroscopy (NFC) pattern in patients diagnosed with both CTD-ILD and non-CTD-ILD to identify microvascular changes and determine the relation between capillaroscopic parameters, clinical variables, and disease-related measurements.

**Patients and methods:**

This cross-sectional study included 95 patients with interstitial lung disease who applied to our Rheumatology and Chest Clinics between September 2021 and July 2023. The patients were divided into two groups based on their diagnosis: non-CTD-ILD (group 1) and CTD-ILD (group 2). Nailfold capillaroscopy was performed.

**Results:**

Ninety-five patients, 49 (51% female, mean age 62.31 ± 11.027 years) in group 1 and 46 (69.6% female, mean age 62.09 ± 10.887 years) in group 2, were included in the study. Abnormal capillary morphologies were both detected in the CTD-ILD group and the non-CTD-ILD groups. In patients with a usual interstitial pneumonia (UIP) pattern on chest computed tomography (CT), tortuosity was higher than in patients with non-specific interstitial pneumonia (NSIP) (*P* = *0.041*), and the proportion of tortuosity increased significantly as the duration of the disease increased (*P* = *0.016*).

**Conclusion:**

Our study highlights capillaroscopic abnormalities alone may not be sufficient to differentiate CTD-ILD (other than systemic sclerosis) from non-CTD-ILD. The presence of NFC abnormalities in non-CTD-ILD may suggest that fibrotic lung disease could potentially play a role in the deterioration of the microvascular structure or abnormal angiogenesis. Our study demonstrated that a multidisciplinary approach, incorporating clinical, morphological, pathological, and serological evaluations, is necessary for interpreting ILD.

**Table:** 

**Key Points** • *Capillaroscopic abnormalities can also be seen in non-CTD-ILD.* • *Capillaroscopy findings do not distinguish the non-Ssc etiology of ILD.* • *Nailfold capillaroscopy may have the potential to serve as a useful tool in predicting prognosis and monitoring the disease progression in patients with idiopathic pulmonary fibrosis (IPF).*

## Introduction

The term “interstitial lung disease” (ILD) refers to a wide range of illnesses marked by interstitial fibrosis and inflammation [1]. ILD is classified based on certain clinical, radiological, and histopathological characteristics. The cause of ILDs is often unknown and referred to as idiopathic. However, in some cases, ILD can be linked to specific environmental factors or an underlying connective tissue disorder [2].

The most common fibrotic ILD is idiopathic pulmonary fibrosis (IPF), which is characterized by a progressive fibrosis and a radiological and/or histological pattern of usual interstitial pneumonia (UIP) [3]. The etiology of ILD includes autoimmune and connective tissue diseases (CTD), sarcoidosis, hypersensitivity pneumonitis (HP), and autoimmune interstitial pneumonia (IPAF), which suggest an underlying autoimmune process but do not meet the criteria for CTD.

HP causes inflammation and fibrosis in the lung parenchyma and small airways. In susceptible individuals, an immune-mediated reaction is triggered by an inhaled antigen, resulting in this condition. There are two types of HP: fibrotic and non-fibrotic. Non-fibrotic HP is characterized by the presence of ground-glass areas that can be found scattered on thoracic computed tomography (CT) sections or may only appear as centrilobular nodules. Fibrotic pulmonary disease can present with various imaging features, including reticulation, traction bronchiectasis, honeycomb formation, centrilobular nodules, or evident air trapping on expiratory images [4].

It is possible for all CTDs to be linked to ILD. As a result, global guidelines suggest that ILD patients should undergo regular assessments for CTD [5]. A rheumatologist plays a crucial role in the multidisciplinary assessment of ILD.

Nailfold capillaroscopy (NFC) is a non-invasive tool that has been approved for diagnosing systemic sclerosis (Ssc) and differentiating primary Raynaud’s phenomenon (RF) from secondary RF. It also has the potential to be used in diagnosing a wider range of CTD. The characteristics of capillaries, both in appearance and number, have been associated with lung problems in CTD. NFC has been suggested as a helpful tool to diagnose CTD in ILD patients. However, its effectiveness in non-rheumatic groups is uncertain due to the limited amount of research conducted [5–7].

The primary aim of this study is to compare the capillaroscopic features of CTD-ILD patients and those without CTD-ILDs. Furthermore, the study intends to examine the correlation between the capillaroscopic findings, the demographic and clinical characteristics of the patients, and the potential role of NFC in the diagnosis and treatment of ILD.

## Material and method

### Study design

This cross-sectional study included 95 ILD patients who applied to our Rheumatology and Chest Clinics between September 2021 and July 2023.

### Settings and patients

Patients between the ages of 18 and 80 were included in the study. Pregnancy, use of long-term oxygen therapy, hypoxic (oxygen saturation level below 90%), coronary artery disease, pulmonary arterial hypertension (systolic pulmonary arterial pressure > 30 in echocardiography), type 2 diabetes mellitus (DM), malignancy, obesity (body mass index (BMI) ≥ 30), patients with active infection, Ssc patients, and individuals who have received immunosuppressive therapy (rituximab, mycophenolate mofetil, azathioprine, cyclophosphamide) diagnoses were excluded from the study.

**Fig. 1:**
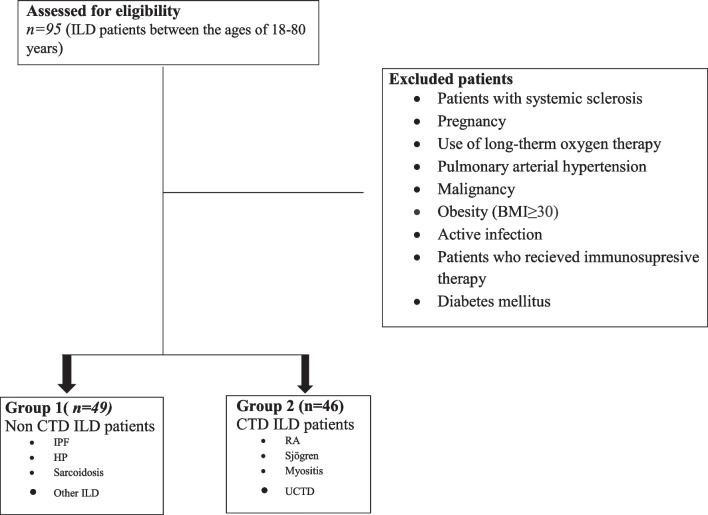
Consort flow diagram

The patients were divided into two groups based on their diagnosis: non-CTD-ILD (group 1) and CTD-ILD (group 2). Age, gender, history of smoking, subgroup diagnosis (IPF, HP, Sarcoidosis, other ILD, rheumatoid arthritis (RA), Sjögren’s (Sjg), myositis, undifferentiated connective tissue diseases (UCTD)), BMI, disease time (for the initial times of the diseases, the beginning of ILD in non-CTD-ILD and the onset of connective tissue disease in CTD-ILD were referenced), and comorbid diseases were recorded (Fig. 1). Patients were questioned about various symptoms including dry mouth/eyes, digital ulcers, uveitis, skin rash, sclerodactyly, telangiectasis, muscle weakness, oral/genital ulcers, inflammatory arthritis, trigger finger, and dyspnea. Oxygen saturation was measured and recorded using a pulse oximeter on the patient’s fingertips. Hematological tests (white blood cell, hemoglobin, platelet), biochemical tests (albumin, calcium, creatinine, glucose), acute phase reactant (CRP, ESH—c-reactive protein, sedimentation), and autoantibody (RF, CCP, ANA, SSA/SSB, Sentromer, Jo-1, SCL-70, ANCA, RNP, anti-dsDNA) results were recorded from the hospital database. At the time of the study visit, forced expiratory volume during the first second (FEV1), forced vital capacity (FVC), and carbon monoxide diffusion capacity (DLCO) were recorded.

### Radiological findings

The pattern of lung involvement on high-resolution CT (HRCT) of the chest was used to determine the radiological findings. Radiologically, the UIP pattern is defined as a honeycomb with or without traction bronchiectasis or bronchiolectasis with subpleural and basal predominant distribution. Typical HRCT features of non-specific interstitial pneumonia (NSIP) include a symmetric lower lobe peribronchovascular distribution with subpleural sparring and superimposed ground-glass opacities. Patterns other than the radiological patterns seen in UIP and NSIP (mosaic attenuation, micronodules, centrilobular nodules, consolidation) and their presence in different distributions (upper or mid-lung) are classified as alternative diagnoses [8]. It was recorded which treatments they had received.

### NFC findings

**Fig. 2:**
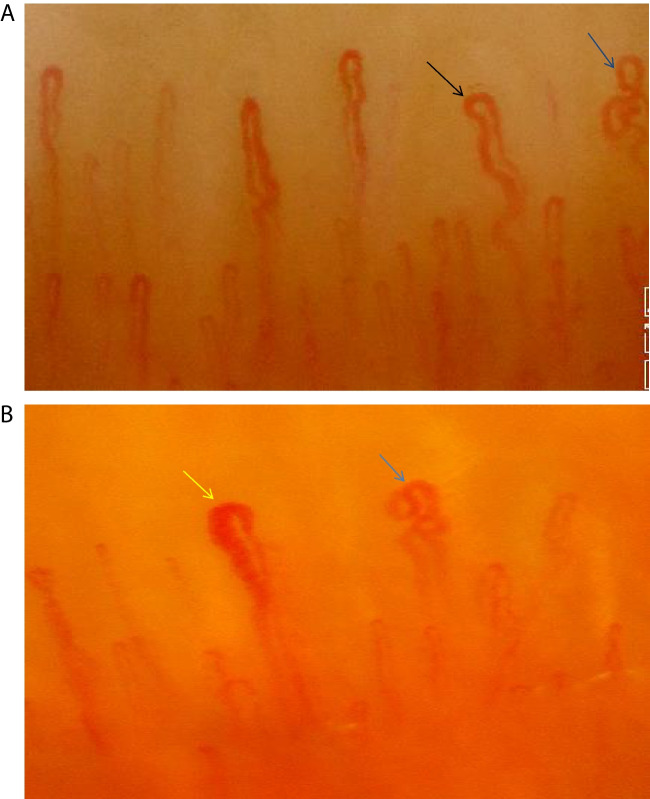
**A** The capillary shown with the blue arrow is an example of neoangiogenesis. The capillary shown with the black arrow is an example of a dilated capillary. **B** The capillary shown with the yellow arrow is an example of a giant capillary. The capillary shown with the blue arrow is an example of neoangiogenesis

During the study, a certified investigator performed nailfold videocapillaroscopy using a digital microscope (Dino-Lite CapillaryScope 200, MEDL4N PRO) and software program (The DinoCapture v2.0 software from AnMo Electronics Corp.). The investigator was careful to remain blinded to the study group to ensure fair results. Before undergoing NFC, participants who had not smoked in the last 30 min were asked to sit in a room with a temperature of around 22–25 °C for at least 15 min. The NFC technique used was of 200 × magnification and involved capturing at least two adjacent areas of 1 mm in the middle of the nailfold finger. The evaluation was done for the second to fifth fingers of both hands. During the assessment, the following parameters were evaluated: capillary density, avascular areas (when more than two capillaries disappear next to each other), dilated capillaries (capillaries with a diameter between 20 and 50 µm) (Fig. 2A), giant capillaries (capillaries with a diameter of 50 µm or more) (Fig. 2B), capillary tortuosity (when there are at least two wavy capillaries in at least two of the eight examined fingers), crossing capillary, hemorrhages, and neoangiogenesis (when there are at least four capillaries in each dermal papilla, and these capillaries are excessively elongated and twisted, originating from a single loop and branching into each other with fine vascular structures) (Fig. 2A, B). To calculate the capillary density, the average area of 1 mm was evaluated across eight fingers. Capillary tortuosity and crossing capillaries were classified as none, below 50%, and above 50%. If tortuosity and crossing were present in at least four of the eight fingers, it was considered above 50%.

### Statistical analysis

Numbers and percentages were used to express categorical variables, while mean, standard deviation, median, and IQR (interquartile range), as appropriate, were used to summarize continuous variables. In order to determine the differences in categorical variables between groups, we utilized either the Pearson chi-square test or Fisher’s exact test. The choice of test was dependent on whether or not an expected value problem was present. These statistical methods were employed to effectively compare and contrast categorical data across different groups. The normal distribution of continuous variables was verified using the Shapiro–Wilk test. In cases where the statistical hypotheses were met, the Student’s *t*-test was used for comparing continuous variables between two groups. If not, the Mann–Whitney *U* test was used instead. To compare continuous variables among multiple groups, we utilized a one-way ANOVA or Kruskal–Wallis test based on whether the statistical hypotheses were met or not. All statistical analyses were conducted using the IBM SPSS Statistics Version 20.0 software package. For all tests, the level of statistical significance considered was 0.05.

SPSS reference: IBM Corp. Released 2011. IBM SPSS Statistics for Windows, Version 20.0. Armonk, NY: IBM Corp.

## Results

One hundred and fourteen patients were evaluated for participation in the study. Five Ssc patients, four patients with hypoxia, six DM patients, and four coronary artery disease patients were excluded from the study. A total of 95 patients, 49 (51% female, mean age 62.31 ± 11.027 years) in group 1 and 46 (69.6% female, mean age 62.09 ± 10.887 years) in group 2, were included in the study.

Table 1 summarizes the comparison of demographic information and clinical characteristics between the two groups. When looking at the lung radiological involvement pattern, the number of patients with the UIP pattern was 33 (67.3%) in the non-CTD-ILD arm and 16 (34.8%) in the CTD-ILD arm. There were 1 patient (2%) in the non-CTD-ILD arm and 7 patients (15.2%) in the CTD-ILD arm in the NSIP pattern. There were more patients with undetermined patterns in the non-CTD-ILD arm than in the CTD-ILD arm (1 (2%), 23 (50%); *P* < 0.001 respectively)). ANA was negative in all patients in group 1, while it was positive in 14 patients (30.4%) in group 2, and as expected, other autoantibodies (SSa/SSb, RNP, Jo-1) were detected positive only in group 2.

**Table 1:** Demographic and clinical characteristics in non-CTD-ILD and CTD-associated ILD

	Non-CTD-ILD patients (*n*: 49) Group 1	CTD-ILD patients (*n*: 46) Group 2	*P*
Female sex, *n* (%)	25 (51)	32 (69.6)	0.065
Age, mean ± SD	62.31 ± 11.027	62.09 ± 10.887	0.532
BMI, mean ± SD	26.21 ± 3.60	27.07 ± 3.46	0.929
Smoker, *n* (%)	25 (56.8)	19 (43.2)	0.412
Subdiagnosis of the group, *n* (%)			
IPF HP Sarcoidosis Other ILD RA Sjögren Myositis UCTD	32 (65.3) 6 (12.2) 9 (18.4) 2 (4.1) - - - -	- - - - 24 (52.2) 11 (23.9) 5 (10.9) 6 (13)	-
Disease duration (month), median (Q1–Q3)	18 (10–25)	27.5 (14–108)	0.509
Treatment, *n* (%)			
Steroids DMARDs Untreatment	24 (49) 0 (0) 25 (51)	16 (34.8) 19 (41.3) 11 (23.9)	< 0.001
Extrapulmonary clinical features, *n* (%)			
Raynaud’s phenomenon Dry mouth/eyes Digital ulcer Sclerodactyly Telangiectasia Muscle wasting Inflammatory arthritis Uveitis Skin Clubbing	0 (0) 1 (2) 0 (0) 0 (0) 0 (0) 0 (0) 0 (0) 0 (0) 2 (4.1) 10 (20.4)	2 (4.3) 19 (41.3) 0 (0) 0 (0) 3 (6.5) 4 (8.7) 31 (67.4) 0 (0) 4 (8.7) 0 (0)	0.232 < 0.001 - - 0.110 0.051 < 0.001 - 0.426 0.001
Laboratory features, median (Q1–Q3)			
CRP (mg/L) ESR (mm/h) Leukocyte (10^9^/L) Hemoglobin (g/dL) Platelet (10^9^/L) Albumin (g/L) Calcium (mg/dL) Uric acid (mg/dL) Glucose (mg/dL) Creatinin (mg/dL)	5 (3–7.1) 22 (15–35) 9.2 (6.9–12.7) 13.4 (12.3–14.7) 264 (228–330) 38.6 (36.9–41.5) 9.5 (9–9.7) 4.6 (3.8–6.1) 106 (90–119) 0.78 (0.6–0.88)	6 (3–12) 25.5 (17–44) 8.2 (6.8–11.6) 12.7 (12.1–14) 263.5 (203–322) 39.2 (36.5–43.4) 9.4 (9.1–9.7) 3.9 (2.9–5.2) 87.5 (81–103) 0.68 (0.58–0.8)	-
Pulmonary function tests, mean ± SD			
FEV1-pred. (%) FVC-pred. (%) FEV1/FVC-pred. (%) DLCO-pred. (%)	87.2 ± 19.24 86.7 ± 20.96 82.1 ± 9.07 54.9 ± 23.63	84.8 ± 20.80 84.3 ± 22.24 84.7 ± 11.20 54.3 ± 23.10	0.587 0.61 0.234 0.906
Radiological pattern, ***n*** (%)			
UIP NSIP Undetermined HP Sarcoidosis	33 (67.3) 1 (2) 1 (2) 5 (10.2) 9 (18.4)	16 (34.8) 7 (15.2) 23 (50) 0 (0) 0 (0)	< 0.001
Echocardiographic parameters, mean ± SD			
EF (%) PAP (mmHg)	61.04 ± 3.64 23.14 ± 3.63	60.63 ± 3.51 23.33 ± 5.32	0.578 0.844

*ILD*, interstitial lung disease; *CTD*, collagen tissue disease; *F*, female; *BMI*, body mass index; *IPF*, interstitial pulmonary fibrosis; *HP*, hypersensitivity pneumonitis; *ILD*, interstitial lung disease; *RA*, rheumatoid arthritis; *UCTD*, undifferentiated connective tissue diseases; *CRP*, C-reactive protein; *ESR*, erythrocyte sedimentation ratio; *FEV1*, forced expiratory volume during the first second; *FVC*, forced vital capacity; *DLCO*, diffusion capacity for carbon monoxide; *EF*, ejection fraction; *PAP*, pulmonary arterial pressure; *UIP*, usual interstitial pneumonia; *NSIP*, non-specific interstitial pneumonia

There was no significant difference in capillaroscopy findings in groups 1 and 2. Capillaroscopic findings are summarized in Table 2. There were a minimum of six and a maximum of ten capillaries in both groups. In the first group, the average capillary count was 8.4 ± 0.9, while in the second group, it was 7.9 ± 0.9. Mean capillary density did not correlate with gender, smoking, BMI, clubbing, clinical findings, or radiological pattern.

**Table 2:** Capillaroscopy features in non-CTD-ILD and CTD-associated ILD

	Group 1 (*n*: 49) Non-CTD-ILD	Group 2 (*n*: 46) CTD-ILD	*P*
Median capillary density, mean ± SD	8.4 ± 0.9	7.9 ± 0.9	0.298
Hemorrhage, *n* (%)	2 (4.1)	6 (13)	0.151
Dilated capillary, *n* (%)	12 (24.5)	16(34.8)	0.637
Giant capillary, *n* (%)	2 (4.1)	1(2.2)	0.999
Avascular area, *n* (%)	-	-	-
Neoangiogenesis, *n* (%)	6 (12.2)	3(6.5)	0.488
Bushy capillary, *n* (%)	2 (4.1)	6(13)	0.151
Ramified capillary, *n* (%)	1(2)	0(0)	0.999
Branching capillary, *n* (%)	1 (2)	0(0)	0.099
Tortuosity, *n* (%)			
None < %50 ≥ %50	17 (34.7) 14 (28.6) 18 (36.7)	15 (32.6) 11 (23.9) 20 (43.5)	0.78
Crossing, *n* (%)			
None < %50 ≥ %50	14 (28.6) 18 (36.7) 17 (34.7)	16 (34.8) 18 (39.1) 12 (26.1)	0.637

The most common qualitative abnormalities detected in our study were tortuosity and crossing of capillaries. Men had a higher prevalence of capillaries that were 50% or more crossed in the NFC compared to women (*P* = 0.047).

There was no association between any of the capillaroscopic features (median capillary density, tortuosity, crossing, dilated capillaries, giant capillaries, hemorrhage, neoangiogenesis) and any of the clinical findings, such as clubbing, Raynaud’s, skin signs, telangiectasia, inflammatory arthritis, dyspnea, muscle weakness or skin signs. However, patients with dry mouth and eyes had statistically higher levels of tortuosity in their NFC findings (11 patients (78.1%); *P* = 0.041).

In our study, we discovered a connection between tortuosity and radiological patterns. The percentage of patients who have ≥ 50 tortuosity was higher in the patients with a UIP pattern on the thorax CT than the patients with NSIP (40.8%, 0.0%; *P* = 0.014 respectively).

When the capillaroscopic features and disease durations were compared between the groups, there was a significant difference in terms of tortuosity between the two groups. CTD-ILD patients (group 2) had longer disease progression times (shown in Table 1). In the study of CTD-ILD patients, it was observed that disease duration was comparatively longer. Additionally, as the duration of the disease increased, the rate of capillaroscopic abnormalities, particularly tortuosity, significantly increased (*P* = 0.016).

Capillary abnormalities defined in the Ssc pattern, including mean capillary count below 7, giant capillaries, hemorrhage, and neoangiogenesis, were detected in the poor prognostic IPF arm (gap point ≥ 3). On the other hand, these capillary patterns were not observed in the good-prognostic IPF arm (gap point < 3). Table 3 summarizes the relationship between capillaroscopic features and gap points.

**Table 3:** Comparison of gap point and capillaroscopic features

	Group 1 (gap points 1–2)	Group 2 (gap points 3–6)	*P*
Patients with median capillary density “6,” *n* (%)	0 (0)	2 (9.1)	0.586
Hemorrhage, *n* (%)	0 (0)	1 (4.5)	0.999
Dilated capillary, *n* (%)	0 (0)	6 (27.3)	0.155
Giant capillary, *n* (%)	0 (0)	1 (4.5)	0.999
Neoangiogenesis, *n* (%)	0 (0)	5 (22.7)	0.287
Tortuosity, *n* (%)			
None < %50 ≥ %50	5 (62.5) 1 (12.5) 2 (25)	6 (27.3) 7 (31.8) 9 (40.9)	0.337
Crossing, *n* (%)			
None < %50 ≥ %50	5 (62.5) 2 (25) 1 (12.5)	6 (27.3) 10 (45.5) 6 (27.3)	0.320

## Discussion

Abnormal NFC findings are mainly found in Ssc patients, which may suggest the presence of microvascular pathologies [9]. European League of Associations for Rheumatology Working Group on Microcirculation in Rheumatic Diseases (EULAR SG MC/RD) has defined scleroderma and non-scleroderma patterns of NFC [10]. The Ssc pattern seen in NFC can also be found in other connective tissue disorders [11].

Lee et al. examined the findings of NFC scans of 81 patients, 31 of whom had CTD-ILD, 18 had IPAF, and 32 had idiopathic interstitial pneumonia (IIP). The researchers found that the NFC scans showed patterns consistent with Ssc. They also observed significant differences between the CTD-ILD and IPAF patients compared to those with IIP [12]. Since Ssc patients were included, the significant difference may be attributed to them. In our study, capillaroscopic features were similar between non-CTD-ILD and CTD-ILD patients. We excluded patients diagnosed with Ssc from our study as NFC patterns are already clearly defined in such patients.

Our study aimed to identify the capillaroscopic features in CTD-ILD and non-CTD-ILD and determine their role in the pathophysiology, diagnosis, and treatment of these diseases. Neoangiogenesis and giant capillaries with the pattern were not found in NFC in IIP patients, according to the study by Lee et al. [12]. In a recently published study by Hysa et al., it was found that patients diagnosed with CTD-ILD showed non-specific abnormalities in capillary morphology and giant capillaries more frequently in Ssc, dermatomyositis, and mixed connected tissue disease (MCTD) compared to other CTDs during the NFC examination [13]. Umashankar et al. conducted a meta-analysis and found that there were notable differences in the prevalence of NFC abnormalities among different CTD-ILDs within the same group [14]. In the study, it was discovered that over 75% of ILD cohorts related to UCTD and MCTD had capillary abnormalities. The ILD groups associated with other connective tissue diseases had less than 50% capillary abnormalities. It appears that the connection between NFC abnormalities and CTD-ILD could differ based on the cause of the underlying CTD. However, in our study, we found non-specific capillary abnormalities (tortuosity, crossing), neoangiogenesis, giant capillaries, hemorrhage, and dilated capillaries in both the CTD-ILD group and the non-CTD-ILD group.

Studies have shown that microvascular damage and pathological angiogenesis are involved in the pathogenesis of ILD such as sarcoidosis and IPF [15, 16]. IPF is a type of chronic and progressive fibrotic ILD characterized by the presence of a UIP pattern. Disorders related to lung fibrosis involve the build-up of fibroblasts and extracellular matrix (ECM). Research suggests that microvascular dysfunction is the main cause of pulmonary fibrosis [17, 18]. In our study, we observed a higher number of tortuous capillaries in the NFC of patients with UIP radiological pattern. In patients who develop pulmonary fibrosis, this finding may be a sign of a microvascular disorder. Clinicians should be aware that in ILD patients, capillaroscopic examination performed periodically after diagnosis may serve as a warning sign of pulmonary fibrosis. Also, our findings indicate that the presence of these findings in NFC suggests that non-CTD-ILD etiology includes microvascular pathology and pathological angiogenesis, even though there was no statistically significant difference between the two groups.

Lee et al. used the GAP score to predict IPF prognosis [19]. The 32 patients diagnosed with IPF for our study were divided into two groups based on their prognosis. Group 1 (gap points 1 and 2) consisted of patients with a good prognosis, while Group 2 (gap points 3 to 6) consisted of patients with a poor prognosis. Although the results were not statistically significant, we saw that capillary anomalies were more common in group 2. The findings suggest that NFC may have the potential to serve as a useful tool in predicting prognosis and monitoring the disease progression in patients with IPF. In addition according to the results of our study, it has been determined that the presence of findings seen in the SSc pattern could also be seen in patients without CTD-ILD and may predict a poor prognosis, especially in patients without CTD-ILD.

In the CTD-ILD arm, we observed an increase in non-specific capillary abnormalities in our study, which was linked to a longer disease course. We observed that as lung fibrosis progressed in the non-CTD-ILD arm, capillaroscopic abnormalities became more prevalent (these abnormalities were less common in the NSIP pattern). As a result, it is expected that as the disease progresses and the fibrosis settles, the number of capillary abnormalities in the non-CTD-ILD arm will rise.

When ILD is first diagnosed, studies recommend conducting NFC to identify any underlying CTD [20]. NFC findings are useful in diagnosing CTD, but we also observed significant abnormal findings such as neoangiogenesis and giant capillaries in ILD patients from the non-CTD group, which could aid in accurate diagnosis of CTD. These capillary abnormalities in NFC may not provide sufficient data to make a definitive decision in the differential diagnosis. It would be beneficial to conduct further studies with a larger number of patients to gain a better understanding of the role of NFC in both the etiology and follow-up of ILD.

Considering the limitations of the study, it is a study conducted in a single center with a relatively small number of patients. Therefore, it cannot be generalized to the entire ILD disease population. Additionally, since ILD is a heterogeneous group of diseases, it is difficult to compare. However, there are few studies on capillaroscopy findings detected; while investigating the etiology of ILD should not always lead to the diagnosis of scleroderma, the patient should be examined as a whole. In addition to examining capillaroscopic features, our study is the first to explore the relationship between gap point and capillaroscopy in IPF patients.

In conclusion, our study highlights the presence of NFC abnormalities in non-CTD-ILD, which may suggest that fibrotic lung disease could potentially play a role in the deterioration of the microvascular structure. Our study demonstrated that a multidisciplinary approach, incorporating clinical, morphological, pathological, and serological evaluations, is necessary for interpreting ILD.

## Abbreviations

ILD: Interstitial lung diseases
CTD-ILD: Connective tissue-associated interstitial lung diseases
NFC: Nailfold capillaroscopy
UIP: Usual interstitial pneumonia
NSIP: Non-specific interstitial pneumonia
IPF: Idiopathic pulmonary fibrosis
HP: Hypersensitivity pneumonitis
IPAF: Autoimmune interstitial pneumonia
Ssc: Systemic sclerosis
RF: Raynaud’s phenomenon
DM: Diabetes mellitus
BMI: Body mass index
RA: Rheumatoid arthritis
Sjg: Sjögren’s
UCTD: Undifferentiated connective tissue diseases
CRP: C-reactive protein
ESH: Sedimentation
FEV1: Volume during the first second
FVC: Forced vital capacity
DLCO: Carbon monoxide diffusion capacity
HRCT: High-resolution CT
EULAR SG MC/RD: European League of Associations for Rheumatology Working Group on Microcirculation in Rheumatic Diseases
IIP: Idiopathic interstitial pneumonia
MCTD: Mixed connected tissue disease
ECM: Extracellular matrix
GAP score: Mortality index in IPF
